# Impact of drug shortages on the work of hospital pharmacists in Japan

**DOI:** 10.1080/20523211.2025.2602285

**Published:** 2025-12-23

**Authors:** Takuru Yasui, Tomoki Takase, Hidefumi Ueno, Yasuhiro Kiko, Fukiko Yamamuro, Taichi Nakashima, Nobuyuki Muroi

**Affiliations:** a Department of Pharmacy, Kobe City Medical Center General Hospital, Kobe, Japan; b Japan Municipal Hospital Association, Tokyo, Japan; c Faculty of Pharmaceutical Science, Kobe Gakuin University, Kobe, Japan; d Department of Pharmacy, Sunagawa City Medical Center, Sunagawa, Japan; e Department of Pharmacy, Fujisawa City Hospital, Fujisawa, Japan; f Department of Pharmacy, Kumamoto City Hospital, Kumamoto, Japan

**Keywords:** Drug shortage, hospital pharmacist, labour cost, questionnaire

## Abstract

**Background:**

Currently, no nationwide reports exist that describe the effects of drug shortages on the work of hospital pharmacists in Japan. Using a questionnaire survey, we evaluated here the impact of recent drug shortages on the work of hospital pharmacists in Japan.

**Methods:**

We distributed a questionnaire to the directors of the pharmacy departments of 853 hospitals belonging to the Japan Municipal Hospital Association. The questionnaire consisted of questions on the situation regarding recent drug shortages at the responding hospitals and the time spent by hospital pharmacists dealing with drug shortages between February 13, 2024, and March 15, 2024.

**Results:**

The proportion of hospitals that answered at least one survey question was 25.7% (219/853). Almost all respondents (98.1% [214/218]) answered that the recent drug shortages negatively affected other practices of the hospital pharmacy. The median time spent dealing with drug shortages in all responding hospitals was 19.5 h of 32 days (including 23 weekdays). The estimated annual labour cost of hospital pharmacists required to deal with the recent drug shortages in the 8,110 hospitals of Japan amounted to 7.1 billion JPY/year (approximately 50 million USD/year).

**Conclusions:**

A survey of directors of hospital pharmacy revealed that recent drug shortages have been a burden on pharmacists at most hospitals, as they have spent a notable amount of time dealing with such drug shortages.

## 1. Background

Drug shortages have been a serious problem worldwide for many years (De Weerdt et al., 2017; Faiva et al., 2021; Kaakeh et al., 2011; Miljković et al., 2025; Obiedalla et al., 2023; Sabogal De La Pava & Tucker, 2022; Shukar et al., 2021; Song et al., 2023; United States Food and Drug Administration, 2023; Uwizeyimana et al., 2021). These shortages have had multiple consequences, including disrupted patient care, treatment delays, increased risk of adverse outcomes, increased healthcare costs, and increased workload and stress for healthcare professionals (Bourneau-Martin et al., 2023, 2025; Kaakeh et al., 2011; Kuruc Poje et al., 2021; McBride et al., 2013; Pauwels et al., 2015; Phuong et al., 2019; Shukar et al., 2021). As a notable example of this, a recent incident led to large-scale drug shortages in Japan. In December 2020, itraconazole tablets, an antifungal agent manufactured by a Japanese generic pharmaceutical company, were contaminated with the sleeping pill rilmazafone, resulting in severe health problems, including hospitalisation or death (Izutsu et al., 2023; Tobita et al., 2022). This incident led to the detection of Good Manufacturing Practice violations of several pharmaceutical companies, which led the Ministry of Health, Labour and Welfare (MHLW) to order the suspension of their operations, resulting in shortages of several medicines (Izutsu et al., 2023; MHLW Japan, 2021).

Although the demand and supply of medicines are generally balanced, drug shortages can occur because of various factors, such as quality problems, shortages of active pharmaceutical ingredients, and the influence of pharmaceutical products from other companies (FPMA Japan, 2024). In such circumstances, even if one pharmaceutical company suspends or restricts the supply of a medicine, the demand for that medicine will exceed its supply in the overall market. Subsequently, other pharmaceutical companies restrict the supply of related medicines to eliminate uneven stock of medicines in each medical institution, called ‘on allocation’ (Kosaka et al., 2023). Consequently, medical institutions face difficulties in obtaining various types of medicines. The numbers of medicines unavailable and allocated in Japan were 743 and 2,400 in August 2021, but 1,556 and 1,795 in February 2025, respectively. Thus, the restricted supply of more than 3,000 medicines has been ongoing for more than three years in Japan (FPMA Japan, 2022, 2024). However, the timeline toward the end of these shortages remains unclear.

To address this national problem, it is important to analyze the current situation of medical institutions, community pharmacies, pharmaceutical companies, administrative divisions, and wholesalers. Additionally, medical institutions should clarify how healthcare providers deal with drug shortages to ensure that patients receive appropriate medications. In particular, hospital pharmacists play a crucial role in addressing drug shortages by gathering information about the medication supply status, considering alternative medicines, and sharing this information with other healthcare providers to minimise the impact on patients (ASHP, 2023; De Weerdt et al., 2017; Shukar et al., 2021). We believe that reporting on the time spent on drug shortages is the best way of objectively reflecting the impact of drug shortages on hospital pharmacists’ work. Some studies have analyzed the impact of drug shortages based on the time spent by hospital pharmacists dealing with such shortages in several countries (De Weerdt et al.; Kaakeh et al., 2011; Pauwels et al., 2015); however, no such study has been reported in Japan. Although drug shortages are a global concern, the situation in Japan has been underreported internationally. Understanding the approaches used by how Japanese hospital pharmacists manage drug shortages can provide useful insights for other countries experiencing similar challenges.

We therefore conducted a nationwide questionnaire survey of pharmacists in municipal hospitals to investigate the impact of recent drug shortages, including those mentioned above.

## 2. Methods

### 2.1. Questionnaire

We developed a questionnaire to analyze the impact of the recent drug shortages on hospital pharmacists **(**after 2021) in Japan. The questionnaire was created using information from previous studies that examined the effects of drug shortages on hospital pharmacists (Baumer et al., 2004; De Weerdt et al., 2017; Kaakeh et al., 2011). No formal validation or pilot testing was conducted; however, the questionnaire items were reviewed by several hospital pharmacists including the co-authors, to ensure clarity and relevance. The questionnaire was sent by email on February 6, 2024, to the directors of the pharmacy department in 853 hospitals belonging to the Japan Municipal Hospital Association (JMHA). A reminder email was sent once during the survey period to encourage responses. The questionnaire consists of two sections, which are, translated into English, presented in Supplemental Materials 1 and 2.

The first section contains questions on the hospital and its situation regarding drug shortages (Supplemental Material 1): the characteristics of the hospital and how it operates (Q1, Q2); the situation at the hospital regarding previous (before 2019) and recent (after 2021) drug shortages (Q3, Q4); the use of information sources regarding medicine supply (Q4); and the time spent by hospital pharmacists dealing with recent drug shortages (Q5). Q4 (7) was an open-ended question, ‘Which medicines significantly increased the workload of hospital pharmacists since the outbreak of recent drug shortages (up to five medicines, after 2021)?’. Additionally, each respondent was required to give reasons for their choices from predefined options (see Supplemental Material 1). In Q5, the respondents were asked to indicate the number of hospital pharmacists who actually spent time dealing with drug shortages and the time spent by the daily detailed classification between February 13 and March 15, 2024 (32 days, including 23 weekdays) (see Supplemental Material 1).

An input table for answering Q5 in Section 1 by date is provided in the second section. Each hospital prospectively self-reported the total time spent dealing with drug shortages during the survey period. Respondents recorded the total duration of shortage-related activities performed by hospital pharmacists each day using the input table provided (Supplemental Material 2). The deadline for responding to the questionnaire was April 26, 2024, after which the data were analyzed.

The Institutional Review Board of Kobe City Medical Centre General Hospital was consulted, and they confirmed that ethical approval was not required due to the design of this study.

### 2.2. Estimated annual labour costs of hospital pharmacists

The annual labour cost of hospital pharmacists required to deal with drug shortages was estimated using the results of the total time in Q5. The average wage of pharmacists in the 2023 basic survey on wage structure conducted by the MHLW was used for this estimation (MHLW Japan, 2024a)*.* The monthly wage of pharmacists was reported to be 388,700 JPY for 165 working hours per month (MHLW Japan, 2024b). The total number of hospitals in Japan was considered to be 8,110 based on the current survey of medical institutions reported by the MHLW (MHLW Japan, 2024a).

### 2.3. Statistical analysis

Continuous data are presented as median (range or interquartile range [IQR]). Data were analyzed using JMP 14.2.0 (SAS Institute Inc., Cary, NC, USA).

## 3. Results

### 3.1. Responding hospitals

In total, 25.7% (219/853) of hospitals responded to at least one survey question (counting 194 general, 17 psychiatric, three university, three general/long-term care, and two convalescent). The number of valid responses varied for each question. Regionally, the proportions of responding hospitals in Honshu, Hokkaido, Kyushu, and Shikoku were 73.1% (160/219), 11.0% (24/219), 9.6% (21/219), and 6.4% (14/219), respectively.

### 3.2. Questionnaire on the negative impact of recent drug shortages

**Figure 1.**
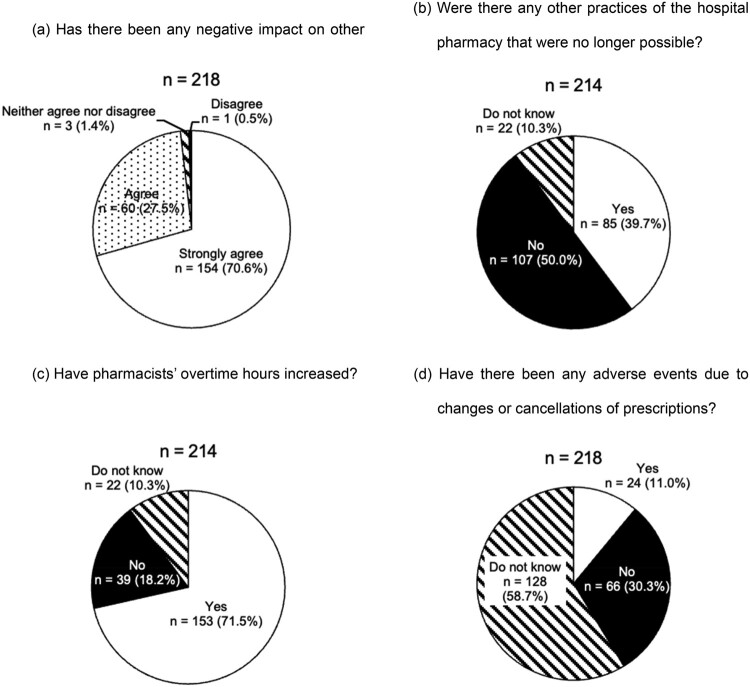
Responses to the questionnaire regarding the negative impact of recent drug shortages.

As shown in Figure 1a, almost all (98.1%) respondents answered that recent drug shortages were related to negative effects on other practices of the hospital pharmacy. Approximately 40% of hospitals reported that some pharmacy practices could no longer be performed because of the increased workload caused by drug shortages (Figure 1b). Moreover, approximately 70% indicated that pharmacists’ overtime hours had increased because of drug shortages (Figure 1c). Although only 11% reported adverse events related to prescription cancellations or changes because of drug shortages, approximately 60% responded that they were unsure whether such events had occurred (Figure 1d).

### 3.3. Questionnaire on the use of and satisfaction with information sources on drug shortages

**Figure 2.**
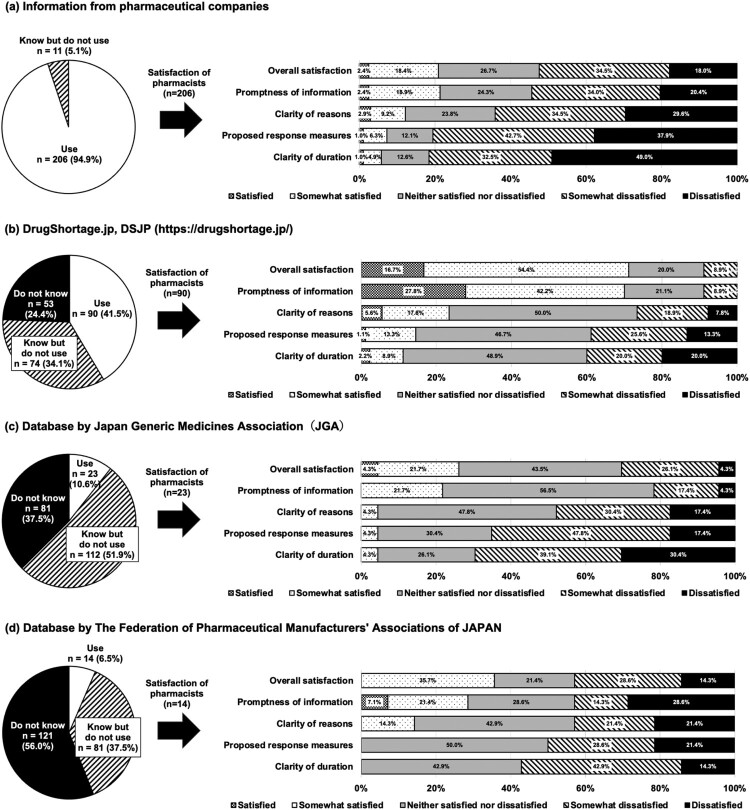
Responses to the questionnaire regarding the use of and satisfaction with information sources on drug shortages.

Figure 2 shows the results of the questionnaire on the use of and satisfaction with four information sources on drug shortages: (a) information from pharmaceutical companies; (b) DrugShortages.jp (a centralised searchable database of medicine supply status, which was originally established by a few clinical pharmacists and is currently operated by the General Incorporated Association asTas); (c) Database of the Japan Generic Medication Association (JGA); and (d) Database of the Federation of Pharmaceutical Manufacturers’ Association of Japan (FPMAJ). Nearly all hospitals (95%) utilised information from pharmaceutical companies, whereas other databases were used less frequently. In particular, the utilisation rates for the Database of the JGA and the Database of the FPMAJ were low. Furthermore, satisfaction with any of these sources was limited; fewer than one-fourth of respondents were satisfied or somewhat satisfied, particularly regarding ‘Clarity of reasons,’ ‘Proposed response measures,’ and ‘Clarity of duration’ (Figure 2).

### 3.4. Medicines that pharmacists at each hospital felt were a heavy burden due to recent drug shortages

Table 1 shows the top 10 medicines that were imposed on heavy workloads for hospital pharmacists owing to recent drug shortages, as well as the most frequent reasons for increasing the workload. Oral acetaminophen was the most commonly reported medicine, followed by intravenous meropenem hydrate, intravenous isotonic sodium chloride solution/prefilled syringe, oral potassium clavulanate/amoxicillin hydrate, oral L-carbocisteine, oral dextromethorphan hydrobromide hydrate, intravenous cefazolin sodium, oral biperiden hydrochloride, intravenous polyethylene glycol-treated normal human immunoglobulin, intravenous urokinase, and oral dimemorfan phosphate. Frequently cited reasons included ‘Frequently prescribed,’ ‘Alternative medicine is not available on the market,’ and ‘Alternative medicine is available but difficult to obtain.’

**Table 1.** Top 10 drugs particularly affecting the workload of hospital pharmacists and the most frequent reasons given*.

Ranking	Name of drug	Dosage form	Number of hospitals, n (%)**	Most frequent reasons
1	Acetaminophen	Oral	68 (32.5%)	(a)
2	Meropenem hydrate	Injection	52 (24.9%)	(b)
3	Isotonic sodium chloride solution/prefilled syringe	Injection	46 (22.0%)	(a)
4	Potassium clavulanate/amoxicillin hydrate	Oral	33 (15.8%)	(c)
5	L-carbocisteine	Oral	30 (14.4%)	(a)
6	Dextromethorphan hydrobromide hydrate	Oral	26 (12.4%)	(b)
7	Cefazolin sodium	Injection	23 (11.0%)	(a)
8	Biperiden hydrochloride	Oral	21 (10.0%)	(a) and (b)
8	Polyethylene glycol-treated normal human immunoglobulin	Injection	21 (10.0%)	(b)
10	Urokinase	Injection	19 (9.1%)	(c)
10	Dimemorfan phosphate	Oral	19 (9.1%)	(a)

*Each respondent could name up to five drugs.

**Percentages refer to a total of 209 responding hospitals.

(a) Frequently prescribed; (b) Alternative medicine is available but difficult to obtain; (c) Alternative medicine is not available on the market

### 3.5. Time spent dealing with recent drug shortages and the estimated annual labour cost

**Figure 3.**
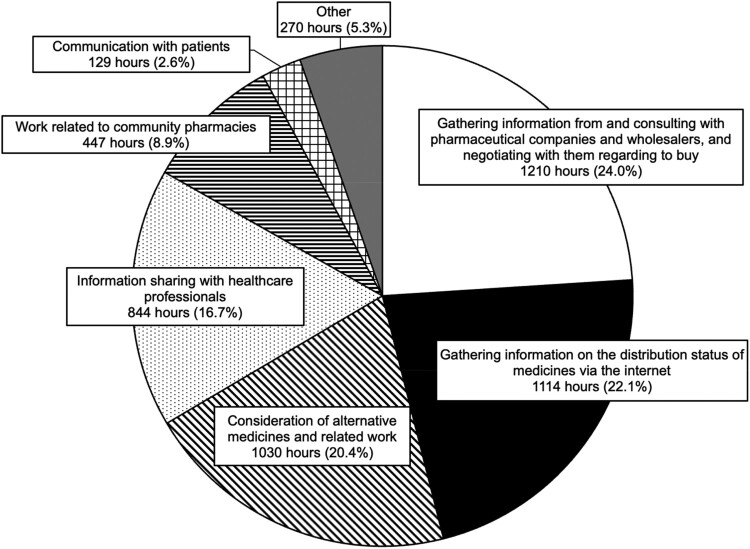
Details of the time spent by hospital pharmacists dealing with recent drug shortages.

The total time spent by hospital pharmacists dealing with recent drug shortages was 5,050 h/162 hospitals between February 13 and March 15, 2024 (32 days, including 23 weekdays). Table 2 lists the aforementioned time according to the number of stratified beds. The median (IQR) time spent dealing with recent drug shortages in all responding hospitals, hospitals with ≤ 99 beds, and hospitals with ≥ 500 beds was 19.5 h (8.9–42.7 h), 7.4 h (4.7–15.2 h), and 38.4 h (19.5–59.7 h), respectively (Table 2). The range of total time reported by individual hospitals varied widely (from 0.5 h to over 242.1 h), reflecting differences in number of beds. The details of the time spent by hospital pharmacists dealing with recent drug shortages are shown in Figure 3. Nearly half of the time was spent on ‘gathering information on the distribution status of medicines via the Internet’ and ‘gathering information from pharmaceutical companies and wholesalers, and negotiating with them regarding the purchase.’

**Table 2.** Time spent by hospital pharmacists dealing with recent drug shortages, stratified by the number of hospital beds.

Number of beds (number of hospitals)	Median (IQR), h
≤ 99 (n = 19)	7.4 (4.7–15.2)
100–199 (n = 29)	16.2 (8.4–27.7)
200–299 (n = 21)	9.5 (6.4–29.0)
300–399 (n = 29)	18.7 (11.1–43.1)
400–499 (n = 23)	34.3 (13.3–63.2)
≥ 500 (n = 41)	38.4 (19.5–59.7)
Total (n = 162)	19.5 (8.9–42.7)

IQR, interquartile range

The estimated annual labour cost for hospital pharmacists required to deal with recent drug shortages at 8,110 hospitals in Japan was 7.1 billion JPY/year.

## 4. Discussion

In this study, we surveyed pharmacists in municipal hospitals belonging to the JMHA on drug shortages occurring after 2021 and their impact on their work. Our data suggest that most hospital pharmacists perceived recent drug shortages as a burden. In fact, hospital pharmacists spent a notable amount of time dealing with drug shortages, including gathering and sharing information and considering alternative medicines. The labour cost of hospital pharmacists across Japan dealing with drug shortages was considerable. To the best of our knowledge, this is the first study to demonstrate the impact of drug shortages after 2021 on the work of pharmacists in municipal hospitals across Japan.

The findings of this study are consistent with the results of previous studies in other countries, wherein the burden of drug shortages on hospital pharmacy operations has been assessed. For example, in a national survey in the United States, it was reported that more than 90% of hospital pharmacists agreed that drug shortages resulted in increased burden or increased cost (Kaakeh et al., 2011). A European study reported that 37% of hospital pharmacists indicated that personnel were severely affected (stressed) by shortages; 22% indicated that personnel were moderately affected (Pauwels et al., 2015). Our results also suggest that recent drug shortages have placed a substantial burden on Japanese hospital pharmacists.

Based on the findings in Figure 1b and c, we speculate that pharmacist overtime hours had increased to maintain other hospital pharmacy practices in many hospitals. The increase in overtime adds to the workload of hospital pharmacists and adversely affects hospital finances because of the additional labour costs. The limited awareness of adverse events among respondents suggests that the true impact of drug shortages on patient safety in Japan is still unclear (Figure 1d). In contrast, drug shortages have been recognised internationally for more than two decades (Baumer et al., 2004), and numerous studies outside Japan have demonstrated their adverse effect on patients’ clinical outcomes, such as treatment changes, medication errors, delayed or denied treatment, prolonged hospitalisation, and even death (Bourneau-Martin et al., 2023, 2025; Kuruc Poje et al., 2021; McBride et al., 2013; Phuong et al., 2019; Shukar et al., 2021). Therefore, future studies are required to clarify the occurrence of adverse events related to drug shortages in Japan.

The survey respondents reported the following medicines as the reason that the workload for hospital pharmacists was particularly increased since the outbreak of recent drug shortages (after 2021): medicines for symptomatic treatment (oral acetaminophen, oral L-carbocisteine, oral dextromethorphan hydrobromide hydrate, and oral dimemorfan phosphate), antibiotics (intravenous meropenem hydrate, oral potassium clavulanate/amoxicillin hydrate, and intravenous cefazolin sodium), intravenous isotonic sodium chloride solution/prefilled syringe, intravenous polyethylene glycol-treated normal human immunoglobulin, oral biperiden hydrochloride and intravenous urokinase (Table 1). The most frequent reason for selecting medicines for symptomatic treatment was ‘Frequently prescribed.’ Medicines for symptomatic treatment are used by many patients. In addition, these medicines have low prices. For example, the generic drug prices in 2024 of acetaminophen 500 mg tablets, L-carbocisteine 500 mg tablets, dextromethorphan hydrobromide 15 mg tablets, and dimemorfan phosphate 10 mg tablets were 1, 9, 8, and 6 JPY/tablet, respectively. In Japan, the government determines the prices of prescription medicine and aims to gradually lower them. Due to low medicine prices, shortages can easily be caused even for essential medicines (Izutsu et al., 2023). Shortages of antibiotics decrease the effectiveness of treatment in patients, as well as increase the prevalence of antibiotic-resistant bacteria (Pandey et al., 2025). The availability of intravenous isotonic sodium chloride solution/prefilled syringe was affected by supply disruptions caused by deviations from manufacturing standards and shortages of a rubber component of the syringe (Otsuka, 2023a, 2023b). Additionally, the supply of intravenous immunoglobulin has decreased as a result of reduced blood collection due to the COVID-19 pandemic and other factors, whereas the number of diseases for which intravenous immunoglobulin is used has increased, raising concerns about a worldwide shortage (Bolcato & Jommi, 2024). Furthermore, the availability of biperiden hydrochloride and intravenous urokinase has been affected by supply disruptions due to difficulties in obtaining active pharmaceutical ingredients from abroad (Mochida et al., 2022; Mitsubishi Tanabe Pharma Corporation, 2023).

The workload of Japanese hospital pharmacists in dealing with recent drug shortages appears broadly comparable to that reported in other countries. Although our survey indicated the median times spent by hospital pharmacists dealing with recent drug shortages was 19.5 h/month (=  4.9 h/week) (Table 2), previous studies reported 9 h/week (IQR, 5–20) in the United States (Kaakeh et al., 2011) and 1.8 h/week (range, 0.7–3.6) in Belgium (De Weerdt et al., 2017). In 166 hospitals of 38 European countries, hospital pharmacists most commonly spent less than 5 h/week dealing with drug shortages; however, some of them spent more than 15 h/week on this task (Miljković et al., 2019). In our survey, the IQR of time spent dealing with drug shortages was 9.0–42.3 h/month (2.0–9.5 h/week), and our result differs only slightly from the findings of previous reports.

In our survey, hospital pharmacists spent a considerable amount of time gathering information related to medicine supply status (Figure 3). When hospital pharmacists obtain information that medicines will be unavailable or will no longer be allocated, they have to investigate the cause of the shortage, determine how long the shortage will last, and consider alternative drugs, if necessary (De Weerdt et al., 2017; Fox & McLaughlin, 2018; Ganio, 2024). Although pharmaceutical companies provide the information, the level of satisfaction associated with this process is low (Figure 2a), and hospital pharmacists are inclined to gather this information themselves. Moreover, for the three sources of information other than pharmaceutical companies, the levels of satisfaction with the clarity of reasons, proposed response measures, and clarity of duration were also low (Figure 2). We speculate that time was spent gathering information because of this situation. Notably, pharmacists in each hospital may gather information in the same way to learn the same content about the situation of medicine supply (De Weerdt et al., 2017). The appropriate and prompt sharing of information from pharmaceutical companies to medical institutions, as well as the development and utilisation of a centralised database collating information on the situation of medicine supply, may aid in reducing the time spent by hospital pharmacists to gather information on drug shortages.

Based on the results of this survey, the labour cost spent dealing with recent drug shortages by hospital pharmacists across Japan was estimated at 7.1 billion JPY/year. This cost is equivalent to 50 million USD, based on the average exchange rate for 2023 (1 USD = 140.62 JPY) (Bank of Japan, Financial Markets Bureau, 2024). Kaakeh et al. reported that the total labour costs for hospital pharmacists, technicians, doctors, and nurses dealing with drug shortages were estimated to be 216 million USD/year nationwide in the United States in 2011 (Kaakeh et al., 2011). Although comparisons are difficult due to fluctuations in the exchange rate (1 USD = 79.73 JPY in 2011) (Bank of Japan, Financial Markets Bureau, 2012), drug shortages clearly have a significant impact on the economy in both Japan and the United States. If the labour costs of other healthcare professionals, such as doctors, nurses, and community pharmacists, are included, the labour costs are expected to be even higher. In addition to the labour costs of healthcare professionals, studies have also reported increased costs due to the purchase of expensive alternative medicines and securing stock, as well as increased patient costs (Fox et al., 2014; Pauwels et al., 2015; Phuong et al., 2019; Shukar et al., 2021;). In the present study, we estimated only the labour costs of hospital pharmacists due to drug shortages. Future studies should focus on the multifaceted impact of drug shortages in Japan.

The findings of this study indicate that drug shortages place a considerable burden on hospital pharmacists, potentially reducing the time available for essential clinical tasks, and indirectly affecting patient care and safety. Therefore, national-level measures to strengthen the pharmaceutical supply chain and ensure the stable supply of medicines are urgently needed. As mentioned above, although the causes of drug shortages are diverse, one proposed solution in Japan involves the government aligning drug prices at a level commensurate with the cost of active pharmaceutical ingredients and manufacturing (Izutsu et al., 2023). In response to recent drug shortages, the MHLW, Japan has been working since 2025 to expand pharmaceutical production capacity and broaden the range of ‘essential drugs’ whose prices are maintained (MHLW Japan, 2025). By further implementing measures to ensure a stable supply of prescription drugs, these efforts will not only help reduce the workload of hospital pharmacists but also contribute to optimising labour costs in healthcare institutions.

This study has some limitations. First, this study was based on the results of a questionnaire survey of municipal hospitals only, and other types of hospitals, such as private or national hospitals, were not included. However, we received responses from hospitals with different functions across the country, including university hospitals, psychiatric hospitals, convalescent hospitals, and hospitals with general and long-term care beds. Therefore, the results of this study reflect the situation of drug shortages in hospitals nationwide. Second, the time spent by hospital pharmacists dealing with recent drug shortages may vary depending on the survey period. The survey period was restricted to the period between February 13 and March 15, 2024; therefore, it is necessary to conduct surveys during other periods. Additionally, the time spent on shortage-related tasks differed among respondents and included extremely long and short times. Notably the survey relied on self-reported data, which may not accurately reflect actual time spent and could differ from more objective measurements such as direct observation. Finally, the survey response rate was not high (25.7%). Empirically, the time spent on drug shortages differs from day to day. For this reason, the survey period was set to 1 month, which is a long period, and the response rate might have been low because of the burden on respondents. Considering that the response rates of similar reports from the United States and Belgium were 27% (Kaakeh et al., 2011) and 12% (De Weerdt et al., 2017), we believe that the response rate in this study is also reasonable.

## 5. Conclusions

A questionnaire survey of directors of hospital pharmacies revealed that recent drug shortages have been a burden on pharmacists in most hospitals, as they spent a notable amount of time dealing with drug shortages.

## Supplementary Material

Supplemental_Material_Clean.docx

## Data Availability

The data underlying this article cannot be shared publicly due to the privacy concerns of the participating hospitals.

## Abbreviations

**Abbreviations:** FPMAJ: Federation of Pharmaceutical Manufacturers’ Associations of Japan; IQR: interquartile range; JGA: Japan Generic Medication Association; JMHA: Japan Municipal Hospital Association; MHLW: Ministry of Health, Labour and Welfare
