# Supplementary material for: Impact of drug shortages on the work of hospital pharmacists in Japan
Source: J Pharm Policy Pract. 2025 Dec 23;19(1):2602285. doi: 10.1080/20523211.2025.2602285 (PMC12777919; doi:10.1080/20523211.2025.2602285)
Supplement: Supplemental_Material_Clean.docx [file JPPP_A_2602285_SM7394.docx]

# Supplemental Material 1.

| **Questionnaire regarding the Impact of Drug Shortages on Hospital Pharmacy Practice** | | | | | | | | | | |
| --- | --- | --- | --- | --- | --- | --- | --- | --- | --- | --- |
| **■ Request regarding input** | | | | | | | | | | |
| ・This survey will examine the impact of the recent drug shortages on hospital pharmacy practice. ・This questionnaire must be completed by one pharmacist representing the pharmacy department. ・Question 5 asks how long pharmacists in your hospital will actually spend dealing with problems caused by drug shortages between 13 February 2024 and 15 March 2024. ・Please distribute the separate sheet (time spent entry form) to pharmacists in your hospital and enter the total time recorded by each pharmacist on the separate sheet. ・In this survey, time spent by non-pharmacists is not included. | | | | | | | | | | |
| **■ Information about the person filling out the form** | | | | | | | | | | |
| (1) Prefecture | | | | | | | | | | |
| (2) Hospital name | | | | | | | | | | |
| (3) Department | | | | | | | | | | |
| (4) Name | | | | | | | | | | |
| (5) Email address | | | | | | | | | | |
| (6) Phone number | | | | | | | | | | |
| **Q1．Overview of your hospital (as of February 1, 2024)** | | | | | | | | | | |
| (1) Hospital type | | | | [**1**. General hospital, **2**. Psychiatric hospital, **3**. Convalescent hospital, **4**. Hospital with general and long-term care beds] | | | | | | |
| (2) Hospital founder | | | | [**1**. Prefecture, **2**. Designated city, **3.** City, **4**. Town/village, **5**. Association, **6**. Other (such as a local independent administrative institution)］ | | | | | | |
| (3) Number of beds | | | | | | | | | | |
| (4) Number of beds most used on a single day in the last year | | | | | | | | | | |
| (5) Number of clinical departments | | | | | | | | | | |
| (6) Number of full-time pharmacists (unit: person) | | | | | | | | | | |
| (7) Number of part-time pharmacists (unit: person, full-time equivalent) | | | | | | [Formula] Full-time equivalent = actual hours worked per week ÷ 40 | | | | |
| (8) Number of pharmacists per 100 beds (unit: person) | | | | | | [Automatic calculation] Total number of pharmacists [(6)+(7)] ÷ (4) × 100 | | | | |
| **Q2．Operation of your hospital** | | | | | | | | | | |
| (1) Main method of prescribing for outpatients | | | | | | [**1**. In-hospital prescription → Go to Q3, **2**. Out-of-hospital prescription → Go to (2)] | | | | |
| ■ If you answered "2. Out-of-hospital prescriptions" in Q2-(1), please answer the following questions. | | | | | | | | | | |
| (2) How to respond to prescription queries by community pharmacists | | | | | | | | | | |
|  | | [ **1**. Community pharmacists ask hospital pharmacists prescription queries, and the hospital pharmacists ask the doctor if necessary.  **2**. Community pharmacists directly ask the doctor prescription queries.  **3**. Others.] | | | | | | | | |
|  |  | **3**. Others [Please specify] | | | | | |  | | |
| (3) Introduction of decision support protocol for prescription queries by community pharmacists | | | | | | | | | | |
| [**1**. Yes (hospital pharmacy-based), **2**. Yes (community pharmacy-based), **3**. Not introduced] | | | | | | | | | | |
|  | | *If your hospital has introduced a decision support protocol for prescription queries by community pharmacists, please answer whether it is hospital pharmacy-based or community pharmacy-based.  The term "hospital pharmacy-based" means that pharmacists at the hospital where the prescribing doctor works use protocols to change prescriptions.  The term "community pharmacy-based" means that community pharmacists use the protocols agreed between the hospital where the prescribing doctor works and the community pharmacy to change prescriptions. | | | | | | | | |
| **Q3. Past drug shortages in your hospital (before 2019)** | | | | | | | | | | |
| (1) Prior to 2019, did you experience drug shortages as you do now? | | | | | | | | | | |
|  | | [**1**. Yes → Go to (2), **2**. No → Go to Q4, **3**. Do not know → Go to Q4] | | | | | | | | |
| ■ If you answered "1. Yes" in Q3-(1), please answer the following question. | | | | | | | | | | |
| (2) Enter the estimated total number of hours per month spent by pharmacists at your hospital in responding to drug shortages before 2019 (leave blank if unknown). | | | | | | | | | | |
| **Q4. Recent drug shortages in your hospital (after 2021)** | | | | | | | | | | |
| (1) Has the increased workload caused by the recent drug shortages had a negative impact on other hospital pharmacy practices? | | | | | | | | | | |
|  | | [ **1**. Strongly agree → Go to (2),  **2**. Agree → Go to (2),  **3**. Neither agree nor disagree → Go to (4),  **4**. Disagree → Go to (4),  **5**. Strongly disagree → Go to (4)] | | | | | | | | |
| ■ If you answered "1. Strongly agree" or "2. Agree" in Q4-(1), please answer the following questions. | | | | | | | | | | |
| (2) Have there been any other hospital pharmacy practices that you are no longer able to do because of the increased workload caused by the recent drug shortages? | | | | | | | | | | |
|  | | [**1**. Yes, **2**. No, **3**. Do not know] | | | | | | | | |
| (3) Have pharmacists’ overtime hours increased because of drug shortages? | | | | | | | | | | |
|  | | [**1**. Yes, **2**. No, **3**. Do not know] | | | | | | | | |
| (4) Have there been any adverse events due to changes or cancellations of prescriptions because of drug shortages? | | | | | | | | | | |
|  | | [**1**. Yes, **2**. No, **3**. Do not know] | | | | | | | | |
| (5) Please answer whether you use the following sources of information on drug shortages and your level of satisfaction with them. | | | | | | | | | | |
|  | | (a) Information from pharmaceutical companies | | | | | | | | |
|  |  |  | [**1**. Use, **2**. Know but do not use, **3**. Do not know] | | | | | | | |
|  |  | ■ If you answered "1. Use" in Q4-(5)-(a), please select your level of satisfaction. | | | | | | | | |
|  |  | Overall satisfaction | | | [**1**. Satisfied **2**. Somewhat satisfied **3**. Neither satisfied nor dissatisfied **4**. Somewhat dissatisfied **5**. Dissatisfied] | | | | | |
|  |  | Promptness of information | | | [**1**. Satisfied **2**. Somewhat satisfied **3**. Neither satisfied nor dissatisfied **4**. Somewhat dissatisfied **5**. Dissatisfied] | | | | | |
|  |  | Clarity of reasons | | | [**1**. Satisfied **2**. Somewhat satisfied **3**. Neither satisfied nor dissatisfied **4**. Somewhat dissatisfied **5**. Dissatisfied] | | | | | |
|  |  | Proposed response measures | | | [**1**. Satisfied **2**. Somewhat satisfied **3**. Neither satisfied nor dissatisfied **4**. Somewhat dissatisfied **5**. Dissatisfied] | | | | | |
|  |  | Clarity of duration | | | [**1**. Satisfied **2**. Somewhat satisfied **3**. Neither satisfied nor dissatisfied **4**. Somewhat dissatisfied **5**. Dissatisfied] | | | | | |
|  |  | (b) DrugShortage.jp, DSJP (https://drugshortage.jp/) | | | | | | | | |
|  |  |  | [**1**. Use, **2**. Know but do not use, **3**. Do not know] | | | | | | | |
|  |  | ■ If you answered "1. Use" in Q4-(5)-(b), please select your level of satisfaction. | | | | | | | | |
|  |  | Overall satisfaction | | | [**1**. Satisfied **2**. Somewhat satisfied **3**. Neither satisfied nor dissatisfied **4**. Somewhat dissatisfied **5**. Dissatisfied] | | | | | |
|  |  | Promptness of information | | | [**1**. Satisfied **2**. Somewhat satisfied **3**. Neither satisfied nor dissatisfied **4**. Somewhat dissatisfied **5**. Dissatisfied] | | | | | |
|  |  | Clarity of reasons | | | [**1**. Satisfied **2**. Somewhat satisfied **3**. Neither satisfied nor dissatisfied **4**. Somewhat dissatisfied **5**. Dissatisfied] | | | | | |
|  |  | Proposed response measures | | | [**1**. Satisfied **2**. Somewhat satisfied **3**. Neither satisfied nor dissatisfied **4**. Somewhat dissatisfied **5**. Dissatisfied] | | | | | |
|  |  | Clarity of duration | | | [**1**. Satisfied **2**. Somewhat satisfied **3**. Neither satisfied nor dissatisfied **4**. Somewhat dissatisfied **5**. Dissatisfied] | | | | | |
|  |  | (c) Database of the Japan Generic Medicines Association (JGA) | | | | | | | | |
|  |  |  | [**1**. Use, **2**. Know but do not use, **3**. Do not know] | | | | | | | |
|  |  | ■ If you answered "1. Use" in Q4-(5)-(c), please select your level of satisfaction. | | | | | | | | |
|  |  | Overall satisfaction | | | [**1**. Satisfied **2**. Somewhat satisfied **3**. Neither satisfied nor dissatisfied **4**. Somewhat dissatisfied **5**. Dissatisfied] | | | | | |
|  |  | Promptness of information | | | [**1**. Satisfied **2**. Somewhat satisfied **3**. Neither satisfied nor dissatisfied **4**. Somewhat dissatisfied **5**. Dissatisfied] | | | | | |
|  |  | Clarity of reasons | | | [**1**. Satisfied **2**. Somewhat satisfied **3**. Neither satisfied nor dissatisfied **4**. Somewhat dissatisfied **5**. Dissatisfied] | | | | | |
|  |  | Proposed response measures | | | [**1**. Satisfied **2**. Somewhat satisfied **3**. Neither satisfied nor dissatisfied **4**. Somewhat dissatisfied **5**. Dissatisfied] | | | | | |
|  |  | Clarity of duration | | | [**1**. Satisfied **2**. Somewhat satisfied **3**. Neither satisfied nor dissatisfied **4**. Somewhat dissatisfied **5**. Dissatisfied] | | | | | |
|  |  | (d) Database of the Federation of Pharmaceutical Manufacturers’ Associations of Japan (FPMAJ) | | | | | | | | |
|  |  |  | [**1**. Use, **2**. Know but do not use, **3**. Do not know] | | | | | | | |
|  |  | ■ If you answered "1. Use" in Q4-(5)-(d), please select your level of satisfaction. | | | | | | | | |
|  |  | Overall satisfaction | | | [**1**. Satisfied **2**. Somewhat satisfied **3**. Neither satisfied nor dissatisfied **4**. Somewhat dissatisfied **5**. Dissatisfied] | | | | | |
|  |  | Promptness of information | | | [**1**. Satisfied **2**. Somewhat satisfied **3**. Neither satisfied nor dissatisfied **4**. Somewhat dissatisfied **5**. Dissatisfied] | | | | | |
|  |  | Clarity of reasons | | | [**1**. Satisfied **2**. Somewhat satisfied **3**. Neither satisfied nor dissatisfied **4**. Somewhat dissatisfied **5**. Dissatisfied] | | | | | |
|  |  | Proposed response measures | | | [**1**. Satisfied **2**. Somewhat satisfied **3**. Neither satisfied nor dissatisfied **4**. Somewhat dissatisfied **5**. Dissatisfied] | | | | | |
|  |  | Clarity of duration | | | [**1**. Satisfied **2**. Somewhat satisfied **3**. Neither satisfied nor dissatisfied **4**. Somewhat dissatisfied **5**. Dissatisfied] | | | | | |
| (6) Please answer whether you use the following consultation service on drug shortages and your level of satisfaction with the service. | | | | | | | | | | |
|  | | (a) Consultation service for the supply of antipyretic analgesic medicines for medical use | | | | | | | | |
|  |  |  | [**1**. Use, **2**. Know but do not use, **3**. Do not know] | | | | | | | |
|  |  | ■ If you answered "1. Use" in Q4-(6)-(a), please select your level of satisfaction. | | | | | | | | |
|  |  | ・Has the problem been resolved by this consultation service? | | | | | | | | |
|  | [**1**. Yes, **2**. No, **3**. Do not know] | | | | | | | | | |
|  | | ・Please select your level of satisfaction with this consultation service. | | | | | | | | |
|  | [**1**. Satisfied, **2**. Somewhat satisfied, **3**. Neither satisfied nor dissatisfied, **4**. Somewhat dissatisfied, **5**. Dissatisfied] | | | | | | | | | |
| (7) Among the medicines that have become difficult to obtain since the outbreak of recent drug shortages (after 2021), please name up to five medicines for which the workload for hospital pharmacists was particularly heavy and the reasons for this. | | | | | | | | | | |
|  |  |  |  |  |  |  |  |  |  |  |
|  | ■ Please select dosage form and reason below. | | | | | | | | | |
|  | Damage form | | | | | | | [**1**. Oral, **2**. Topical, **3**. Injection] | | |
|  | Reason | | | | | | | [ **1**. Alternative medicine is not available on the market,  **2**. Alternative medicine is available but difficult to obtain,  **3**. Frequently prescribed,  **4**. Difficult to obtain for a long time,  **5**. Preparation or dispensing in the pharmacy department that was not previously required has become necessary,  **6**. Other] | | |
|  |  | | | | | |  | | | |
|  | Drug Name | | | | | | | Form | Reason | Other [Please specify] |
|  |  | | | | | | |  |  |  |
|  |  | | | | | | |  |  |  |
|  |  | | | | | | |  |  |  |
|  |  | | | | | | |  |  |  |
|  |  | | | | | | |  |  |  |
| **Q5. The number of pharmacists and the time spent by your hospital pharmacists dealing with the recent drug shortages** | | | | | | | | | | |
| Please answer the number of pharmacists in your hospital who actually spent time dealing with the drug shortages and the time (unit: minute) on a daily basis from February 13, 2024, to March 15, 2024.  Please distribute the separate time spent entry form to the relevant pharmacists and record the total time after each pharmacist has recorded it. | | | | | | | | | | |
| ■ The number of pharmacists with a workload related to drug shortages in your hospital (unit: person) | | | | | | | | | | |
| (1) Gathering information on the distribution status of medicines via the Internet | | | | | | | | | | |
|  | | (e.g., time spent gathering information from the Internet, including pharmaceutical company websites and medicine information databases) | | | | | | | | |
| (2) Gathering information from pharmaceutical companies and wholesalers, and negotiating with them regarding the purchase | | | | | | | | | | |
|  | | (e.g., time spent gathering information directly from pharmaceutical companies and wholesalers, and time spent discussing or negotiating to buy) | | | | | | | | |
| (3) Consideration of alternative medicines and related work | | | | | | | | | | |
|  | | (e.g., time spent gathering information on alternative medicines, time spent consulting with doctors, time spent updating the database in relation to medicines) | | | | | | | | |
| (4) Information sharing among healthcare professionals | | | | | | | | | | |
|  | | (e.g., time spent sharing information with doctors or pharmacists in the hospital, time spent preparing documents used for such information sharing) | | | | | | | | |
| (5) Work related to community pharmacies | | | | | | | | | | |
|  | | (e.g., time spent responding to prescription queries by community pharmacists, time spent consulting and sharing information with community pharmacists) | | | | | | | | |
| (6) Communication with patients | | | | | | | | | | |
|  | | (e.g., time spent explaining medication to patients, time spent responding to inquiries by patients) | | | | | | | | |
| (7) Other | | | | | | | | | | |

# Supplemental Material 2.

| Hospital name | | | |  | | | |
| --- | --- | --- | --- | --- | --- | --- | --- |
| Number of pharmacists with a workload related to the recent drug shortages in your hospital | | | |  | | | |
|  | | | | | | | |
| ■Total time spent by your hospital pharmacists actually dealing with drug shortages by the following items by date (unit: minutes)  [Items]  (1) Gathering information on the distribution status of medicines via the Internet  (2) Gathering information from and consulting with pharmaceutical companies and wholesalers, and negotiating with them regarding the purchase  (3) Consideration of alternative medicines and related work  (4) Information sharing with healthcare professionals  (5) Work related to community pharmacies  (6) Communication with patients  (7) Other | | | | | | | |
| Date／Item | 1 | 2 | 3 | 4 | 5 | 6 | 7 |
| 13-Feb-2024 |  |  |  |  |  |  |  |
| 14-Feb-2024 |  |  |  |  |  |  |  |
| 15-Feb-2024 |  |  |  |  |  |  |  |
| 16-Feb-2024 |  |  |  |  |  |  |  |
| 17-Feb-2024 |  |  |  |  |  |  |  |
| 18-Feb-2024 |  |  |  |  |  |  |  |
| 19-Feb-2024 |  |  |  |  |  |  |  |
| 20-Feb-2024 |  |  |  |  |  |  |  |
| 21-Feb-2024 |  |  |  |  |  |  |  |
| 22-Feb-2024 |  |  |  |  |  |  |  |
| 23-Feb-2024 |  |  |  |  |  |  |  |
| 24-Feb-2024 |  |  |  |  |  |  |  |
| 25-Feb-2024 |  |  |  |  |  |  |  |
| 26-Feb-2024 |  |  |  |  |  |  |  |
| 27-Feb-2024 |  |  |  |  |  |  |  |
| 28-Feb-2024 |  |  |  |  |  |  |  |
| 29-Feb-2024 |  |  |  |  |  |  |  |
| 1-Mar-2024 |  |  |  |  |  |  |  |
| 2-Mar-2024 |  |  |  |  |  |  |  |
| 3-Mar-2024 |  |  |  |  |  |  |  |
| 4-Mar-2024 |  |  |  |  |  |  |  |
| 5-Mar-2024 |  |  |  |  |  |  |  |
| 6-Mar-2024 |  |  |  |  |  |  |  |
| 7-Mar-2024 |  |  |  |  |  |  |  |
| 8-Mar-2024 |  |  |  |  |  |  |  |
| 9-Mar-2024 |  |  |  |  |  |  |  |
| 10-Mar-2024 |  |  |  |  |  |  |  |
| 11-Mar-2024 |  |  |  |  |  |  |  |
| 12-Mar-2024 |  |  |  |  |  |  |  |
| 13-Mar-2024 |  |  |  |  |  |  |  |
| 14-Mar-2024 |  |  |  |  |  |  |  |
| 15-Mar-2024 |  |  |  |  |  |  |  |
